# A meta-analysis for the diagnostic accuracy of SelectMDx in prostate cancer

**DOI:** 10.1371/journal.pone.0285745

**Published:** 2024-02-08

**Authors:** Hanting Wu, Yanling Wu, Peijie He, Juan Liang, Xiujuan Xu, Conghua Ji

**Affiliations:** 1 School of Public Health, Zhejiang Chinese Medical University, Hangzhou, China; 2 Institute of Medicine, Sahlgrenska Academy at University of Gothenburg, Gothenburg, Sweden; 3 Tongde Hospital of Zhejiang Province, Hangzhou, China; 4 The First Affiliated Hospital of Zhejiang Chinese Medical University, Hangzhou, China; University of Buea, CAMEROON

## Abstract

To overview the diagnostic accuracy of SelectMDx for the detection of clinically significant prostate cancer and to review sources of methodologic variability. Four electronic databases, including PubMed, Embase, Web of Science, and Cochrane Library were searched for eligible studies investigating the diagnostic value of SelectMDx compared with the gold standard. The pooled sensitivity, specificity, and positive and negative predictive values were calculated. Included studies were assessed according to the Standards for Quality Assessment of Diagnostic Accuracy Studies 2 tool. The review identified 14 relevant publications with 2579 patients. All reports constituted phase 1 biomarker studies. Pooled analysis of findings found an area under the receiver operating characteristic analysis curve of 70% [95% CI, 66%-74%], a sensitivity of 81% [95% CI, 69%-89%], and a specificity of 52% [95% CI, 41%-63%]. The positive likelihood ratio was 1.68, and the negative predictive value is 0.37. Factors that may influence variability in test results included the breath collection method, the patient’s physiologic condition, the test environment, and the method of analysis. Considerable heterogeneity was observed among the studies owing to the difference in the sample size. SelectMDx appears to have moderate to good diagnostic accuracy in differentiating patients with clinically significant prostate cancer from people at high risk of developing prostate cancer. Higher-quality clinical studies assessing the diagnostic accuracy of SelectMDx for clinically significant cancer are still needed.

## Introduction

Prostate cancer (PCa) is a global health problem for men [1]. In 112 countries, PCa is the most commonly diagnosed cancer in the men population in 2020 [2]. It is estimated that globally 1,414,259 new cases of PCa lead to 375,304 deaths in 2020 [2]. Even in Asian countries, which were considered to have a lower incidence than Western countries, the incidence of PCa has risen rapidly [3].

Currently, the serum prostate-specific antigen (PSA) is clinically widespread for PCa screening and PCa is often diagnosed following the elevation of PSA [4]. In the United States, mortality due to PCa has substantially decreased since the advent and widespread of the PSA test [5]. However, as an early detection biomarker, PSA was not cancer-specific [6]. Age, prostatitis, benign prostate conditions, and medications such as nonsteroidal anti-inflammatory drugs (NSAIDs), statins, and thiazide diuretics also have impacts on the PSA value [6–8]. Data from one randomized study from Europe showed that when cutoffs were defined between 2.5 and 4.0 μg/L, the false-positive rate of PSA test results was approximately 80% [9]. Compliance with the following prostate biopsy was also not so high in patients with positive PSA test results. PSA screening is controversial and there is growing attention to the potential problems of overdiagnosis and treatments caused by PSA screening for prostate cancer [10]. Even in 2008, after PSA was introduced and continued to be common practice for several years, the U.S. Preventive Services Task Force (USPSTF) recommended against routine screening [11–13]. As the gold standard for PCa diagnosis, prostate biopsies may cause physical distress, pain, and a 4% risk of a serious infection or bleeding, whereas only approximately 25% to 40% of patients who underwent biopsy will be diagnosed with prostate cancer [14–16]. Therefore, there is a need for a non-invasive tool with higher diagnostic accuracy for PCa.

SelectMDx is a novel urine-based risk score, which combines urinary biomarkers homeobox C6 (HOXC6) and distal-less homeobox 1 (DLX1) with traditional clinical factors such as age, PSA, digital rectal examination (DRE), prostate volume, and family history of PCa to assess the probability of risk of suffering clinical significant PCa (Gleason score ≥ 7 or Grade group ≥ 2) [17–19]. This risk score could reach a sensitivity of 96% and a specificity of 53% in the validation cohort [20]. The SelectMDx may have the potential to become a promising diagnostic tool for clinically significant PCa.

Several trials have investigated the diagnostic accuracy of SelectMDx for clinically significant PCa, but there are large differences in sensitivity and specificity values among individual studies [20, 21]. Therefore, we performed a systematic review and meta-analysis to comprehensively assess the diagnostic value of SelectMDx for clinically significant PCa and evaluate the quality of existing evidence.

## Method

### Protocol and registration

This study followed the Preferred Reporting Items for Systematic Reviews and Meta-analyses (PRISMA) guidelines for reporting (2020-version) [22]. The protocol for this meta-analysis was registered in PROSPERO (CRD42022338323).

### Search strategy and selection criteria

We searched electronic databases such as PubMed, Embase, Web of Science, and Cochrane Library to identify studies reporting data for SelectMDx for the detection of PCa until April 16, 2023. “SelectMDx”, “2-Gene mRNA urine test”, “urinary molecular biomarker-based risk score”, “HOXC6”, “DLX1”, “prostate cancer”, “prostate neoplasms”, and “prostatic cancer” were used as keywords. The detailed search strategies are provided in the S1 File. References of articles identified were also searched manually. There was no restriction on the publication date and language.

All studies evaluating the sensitivity and specificity of SelectMDx, which used prostate biopsy (the gold standard diagnosis for PCa) as a reference, were considered eligible. Studies with incomplete information for data analysis were also not included.

### Study screening

All potential references were imported into Endnote X9 (Clarivate Analytics) and duplicates were removed using the software. Two independent reviewers screened each citation and abstract to identify eligible studies. Full texts of these studies were also reviewed if necessary. Possible disagreements between reviewers were resolved by discussion with a third reviewer.

### Data extraction and quality assessment

The following basic information was extracted from included studies: author, year, study design, the definition of high-grade PCa, cut-off value, and patient characteristics. Two researchers independently extracted the information from the included full–text papers and the discrepancy was resolved by consensus with a third researcher. Sample size and the number of true positives (TP), true negatives (TN), false positives (FP), and false negatives (FN) were also obtained for data analysis. If different cut-off values were reported in the same study, data with higher sensitivity was extracted and analyzed in this meta-analysis.

The Quality Assessment of Diagnostic Accuracy Studies–2 (QUADAS-2) tool [23], which focuses on the bias and applicability of study results, was used in the quality assessment of included studies. The QUADAS-2 consists of a Quality assessment conducted independently by two reviewers and a third reviewer was consulted to resolve any possible disagreements.

### Data analysis

For each study, TP, TN, FP, and FN were collected and entered into a standard two-by-two table to calculate sensitivity, specificity, positive likelihood ratios (PLR), and negative likelihood ratios (NLR) [24, 25]. A summary ROCs curve and the respective area under the curve were also generated. The I^2^ statistic was used to evaluate heterogeneity between studies. Statistically significant heterogeneity was defined as P<0.05 or I^2^ greater than 50% [26, 27].

When significant heterogeneity was detected, a meta-regression analysis was performed to explore the source of heterogeneity by subgrouping the following possible causes of heterogeneity: 1) median PSA level (>4 ng/ml or ≤4 ng/ml), while the American Cancer Society (ACS) recommends considering biopsy for men with PSA >4 ng/ml [28]; 2) cut-off value, whether a definite cut-off value was provided; 3) subjects, whether only patients previously diagnosed with low-risk PCa or patients suspected with PCa were included; 4) sample size (≤50 or >50).

Statistical analysis was performed using Stata (Version 16.0, Stata Corporation) with metan and midas modules.

## Results

### Study selection

**Fig 1:**
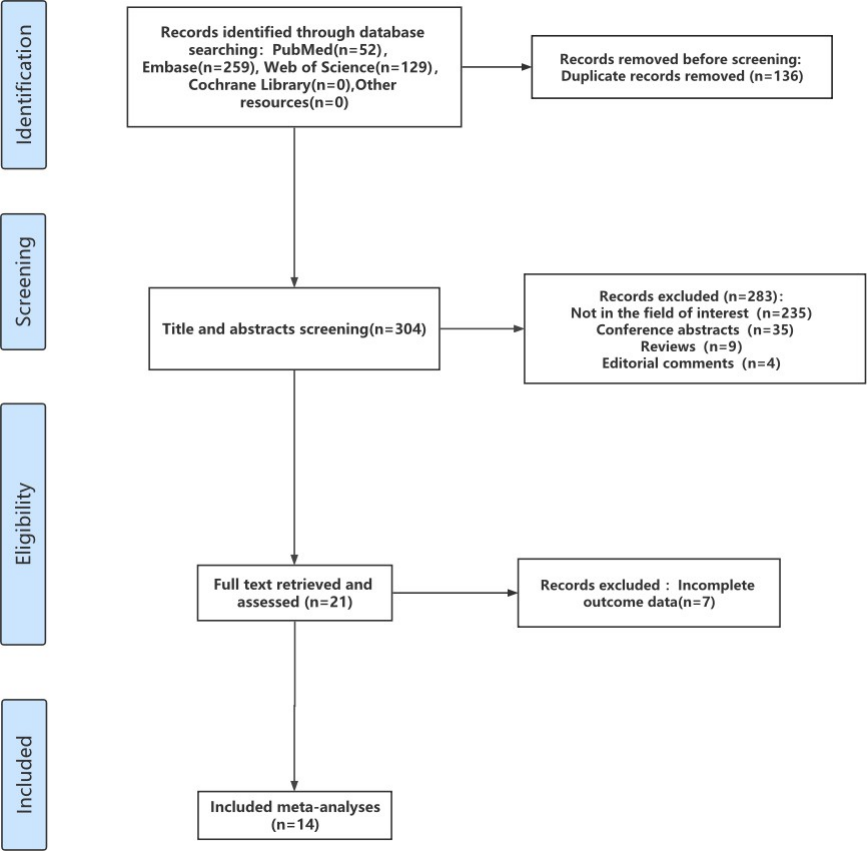
Flow chat.

The literature search identified 440 articles. 136 duplicates were removed using Endnote X9.1(EndNote, Clarivate Analytics). After screening the titles and abstracts, 283 articles were excluded for the following reasons: 235 articles that were not in the field of interest, 35 conference abstracts, 9 reviews, and 4 editorial comments. 21 full texts were reviewed and 7 articles were excluded because of incomplete data for analysis. Finally, 14 studies investigating the diagnostic accuracy of SelectMDx for clinically significant PCa [29–42], with a total of 2579 patients, were included in this meta-analysis. The study selection process is described in Fig 1.

### Characteristics of included studies

Table 1 summarizes the key characteristics of included studies and participants. All studies were conducted between 2019 and 2022, and 40% of the studies were in 2021. Two studies included only patients with low-risk PCa. Other studies all recruited patients suspected of PCa for various reasons. The number of included patients who underwent SelectMDx and prostate biopsy ranged from 12 to 916. Twelve studies were prospective studies and two were retrospective. One of the studies was a multicenter trial in which men from the Netherlands, France, and Germany were enrolled. Four of the studies were conducted in the US and three were from Italy. The remaining studies were from Spain (2/14), the Netherlands (1/14), Germany (1/14), the UK (1/14), and France (1/14).

**Table 1:** Characteristics of included studies.

Author	Year	Study Design	Location	Sample Size	Enrolment Criteria	Definition of High-grade PCa	Prostate Biopsy Procedure	Median Age (Range)	Median PSA(IQR)	Prevalence of Prostate Cancer	Date of Patient Selection	Cut-off Value of SelectMDx
Busetto [29]	2021	Prospective	Italy	52	Men who were scheduled for initial prostate biopsy, based on elevated total PSA level (> 3 ng/ml confirmed) or abnormal digital rectal examination (DRE)	Gleason score ≥ 7(Grade group≥ 2)	Transrectal ultrasound guided biopsy (TRUSGB)	67(44–79)	5.9(1.9–19.9)	32.69%	March 2018 to September 2019	/
Cussenot [41]	2022	Prospective	UK	66	Men with a high genetic risk (familial or personal history of cancers or a predisposing germline variant)	Prostate biopsy with International Society of Urological Pathology(ISUP) grade >1	TRUSGB	59(39–75)	6.7(4.9–10.3)	46.27%	2010 to 2018	/
Haese [30]	2019	Prospective	Netherlands France Germany	916	Men underwent an initial prostate biopsy for suspected PCa	Gleason score ≥ 7(Grade group≥ 2)	TRUSGB	64 (59–69)^*^	5.4(4.1–7.2)	45.73%	December 2007 to December 2014	-2.8
Hendriks [31]	2021	Prospective	Netherlands	599	Men aged 50–75 years with a PSA level of ≥3.0 ng/ml	Gleason score ≥ 7(Grade group≥ 2)	TRUSGB	65 (59–68)^*^	7.4(5.3–11.7)	53.59%	July 2009 to July 2014	-2.8
Lendinez-Cano [32]	2021	Prospective	Spain	163	Men underwent an initial prostate biopsy for suspected PCa	Gleason score ≥ 7(Grade group≥ 2)	TRUSGB and targeted biopsy	62 (57–67)	5.21(4.26–6.31)	44.17%	May 2019 to February 2020	15%
Maggi [33]	2021	Prospective	Italy	310	Men with elevated total PSA level(>3 ng/mL confirmed) and/or abnormal DRE.	Gleason score ≥ 7(Grade group≥ 2)	TRUSGB and targeted biopsy	65(44–79)	6.1(1.0–19.5)	33.55%	March 2018 to September 2019	/
Morote [34]	2022	Prospective	Spain	62	Men with a lesion with a PI-RADS v.2 score of 3	/	Guided and systematic biopsies	/	/	29.03%	May 2019 to April 2021	13%(-2.8)
Pepe [35]	2020	Prospective	Italy	45	Men with very low-risk PCa	Gleason score ≥ 7(Grade group≥ 2)	TRUSGB and targeted biopsy	66(60–73)	/(0–10)^#^	84.44%	July 2015 to July 2018	26%
Rahnama’i [36]	2021	Prospective	Germany	39	Men underwent a mpMRI before their transrectal prostate biopsies and had additionally undergone a liquid biopsy test (SelectMDx).	Gleason score ≥ 7(Grade group≥ 2)	TRUSGB	66 (45–77)	/(0.5–66.78)	100.00%	July 2018 to November 2020	50%
Roumiguié [37]	2020	Prospective	France	117	Men received image-guided biopsy (IGB) after MRI	Gleason score ≥ 7(Grade group≥ 2)	Image-guided biopsy (IGB)	65 (63–67)	7.0(6.5–8.0)	46.15%	/	-2.8
Sessine [38]	2021	Prospective	US	12	Men aged 35−75 years with pathogenic germline or family history of prostate, breast, ovarian, or pancreatic cancer.	Gleason score ≥ 7(Grade group≥ 2)	TRUSGB and targeted biopsy.	61.5(35–71)	2.96	33.33%	April 2017 to January 2020	27.5
Shore [39]	2019	Retrospective	US	80	Men received a SelectMDx test while under consideration for a possible initial prostate biopsy.	Gleason score ≥ 7(Grade group≥ 2)	/	67(62–72)^*^	5.1(3.8–7.1)^#^	38.75%	May 2016 to April 2017	/
Stanton [42]	2022	Retrospective	US	68	Men who underwent transperineal mapping biopsy (TPMB) after TRUS biopsy	Gleason score ≥ 7(Grade group≥ 2)	TPMB	63(57–68)^*^	5.1(3.0–7.6)	85.29%	/	/
Wysock [40]	2020	Prospective	US	50	Men with an elevated PSA and no prior biopsy evidence of PCa	Gleason score ≥ 7(Grade group≥ 2)	TRUSGB and targeted biopsy.	63(52–74)	5.25 (3.8–8.13)	52.00%	November 2018 to April 2019	7.5%/12%

*Median Age (IQR)

#Median PSA (Range)

In 85.71% (12/14) of included studies, the median age of participants ranged from 61.5–67. A total of 11 studies reported the median PSA level of participants. Except for one study, the median PSA level was 2.96 ng/mL. For the other ten investigations, the overall median PSA range was 5.1–7.4 ng/mL. The prevalence of PCa in each study ranged from 29.03% to 100%. Cut-off values of SelectMDx were reported in 64.29% of included studies and a cut-off value of -2.8 (This value is equivalent to the probability of 13% that following prostate biopsy would identify high-grade PCa) was used in four studies.

### Quality assessment

Assessment of the methodological quality of the 14 included studies with the QUADAS-2 tool indicated that most were of moderate to high quality, with concerns arising mainly about patient selection, flow and timing, and index text (Table 2) [43]. For the patient selection domain, a high or unclear risk of bias was seen in 42.86% (6/14) of QUADAS-2 assessments, mostly related to including only patients with previous PCa diagnoses confirmed by transrectal biopsy. For the index test domain of applicability, 35.71% (5/14) of assessments were considered an unclear risk of bias because it was unknown whether the cut-off values for classifying SelectMDx test results as positive or negative were pre-specified. Given only studies in which prostate biopsy was used as a reference were included, in the reference standard domain, we judged the risk of bias as low in all of the assessments.

**Table 2:** Quality assessment of included studies.

		Risk of Bias				Applicability Concerns		
Author	Year	Patient Selection	Index Test	Reference Standard	Flow and Timing	Patient Selection	Index Test	Reference Standard
Busetto [29]	2021	?	L	L	H	L	L	L
Cussenot [41]	2022	L	?	L	L	L	?	L
Haese [30]	2019	L	L	L	?	L	?	L
Hendriks [31]	2021	L	L	L	L	L	L	L
Lendinez-Cano [32]	2021	H	L	L	L	H	L	L
Maggi [33]	2021	L	L	L	L	L	L	L
Morote [34]	2022	?	L	L	L	?	L	L
Pepe [35]	2020	H	L	L	L	H	L	L
Rahnama’i [36]	2021	L	?	L	L	L	?	L
Roumiguié [37]	2020	L	?	L	L	L	?	L
Sessine [38]	2021	?	L	L	L	L	L	L
Shore [39]	2019	L	L	L	L	L	L	L
Stanton [42]	2022	L	?	L	L	L	?	L
Wysock [40]	2020	?	L	L	L	L	L	L

L: Low risk; H: High risk

### Diagnose performance

#### Pooled sensitivity and specificity

**Fig 2:**
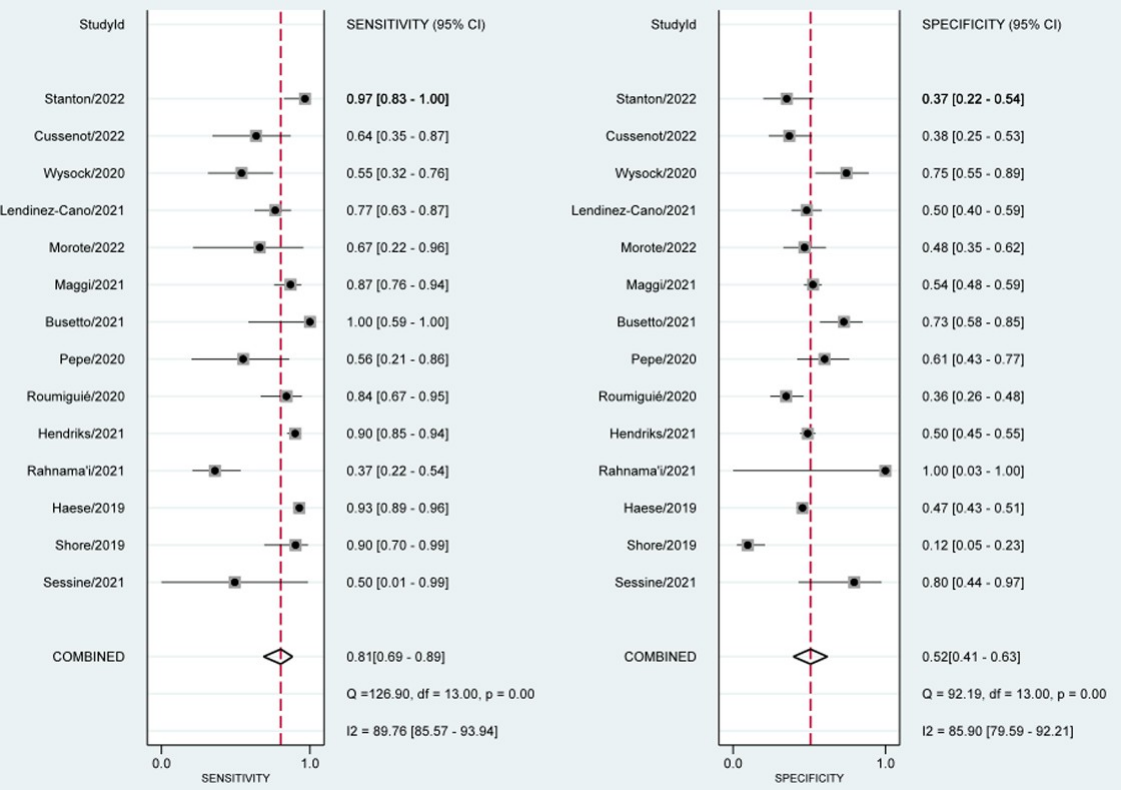
Pooled sensitivity and specificity of SelectMDx.

**Fig 3:**
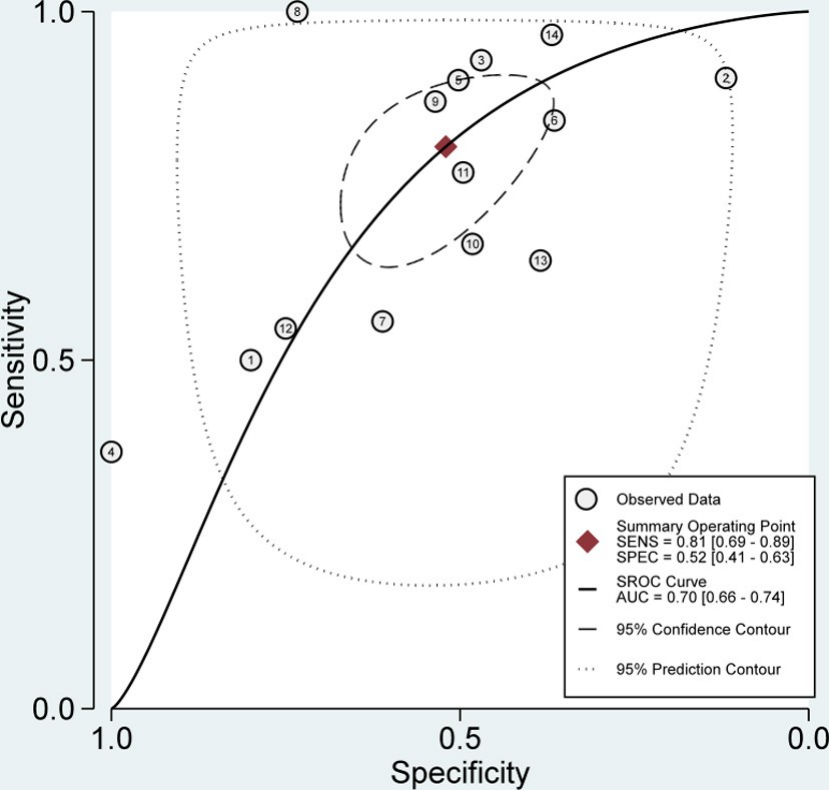
SROC with prediction & confidence contours.

Figs 2 and 3 show the pooled evidence of included studies. The sensitivity of SelectMDx for the detection of clinically significant PCa ranged from 37% to 100%, whereas the specificity ranged from 12% to 100%. The bivariate random-effects meta-analysis of the 14 studies showed a pooled sensitivity of 81% [95% CI, 69%-89%] and a pooled specificity of 52% [95% CI, 41%-63%] for SelectMDx (Fig 2). Forest plots demonstrated a high degree of heterogeneity for both sensitivity and specificity estimates, with I^2^ indexes of 89.76% and 85.90%, respectively (Fig 2). The positive likelihood ratio (PLR) and negative likelihood ratio (NLR) of SelectMDx were 1.68 [95% CI, 1.38–2.05] and 0.37 [95% CI: 0.24–0.57], respectively. The overall diagnostic odds ratio (DOR) was 4.51 [95% CI: 2.64–7.72]. Pooled receiver operating characteristic analysis of all included studies in a pooled area under the curve (AUC) was 70% [95% CI, 66%-74%] (Fig 3).

#### Clinical utilization

**Fig 4:**
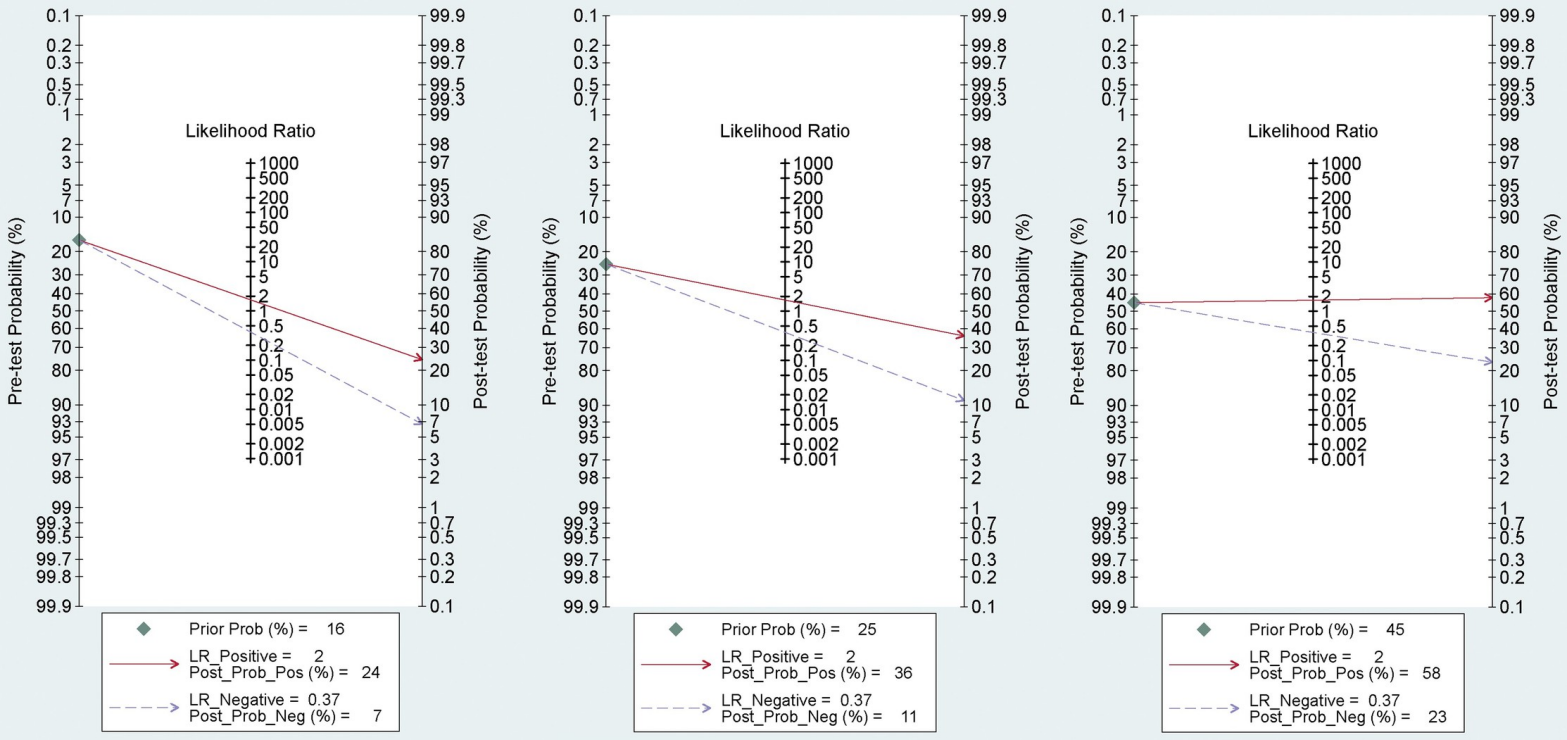
Fagan plot of SelectMDx.

Fagan plot analysis was used to visualize the diagnostic performance of SelectMDx (Fig 4). In included studies, patients with a PSA level of ≥3.0 ng/ml or abnormal digital rectal examination (DRE) were enrolled. Pretest probability in this meta-analysis was defined as the probability that the patient had clinically significant PCa before testing. In patients with elevated total PSA levels (≥ 3 ng/ml), the prevalence of clinically significant PCa varies with PSA levels [44, 45]. It is assumed that the pretest probability of diagnosing clinically significant PCa is 16%. The analysis of the Fagan plot testified that SelectMDx could provide certain informative utility for diagnosing clinically significant PCa with a probability increased to 24% of correct diagnosis following a “positive” measurement and lowering the probability of disease to 7% following a “negative” measurement. When the pre-test probability for diagnosing clinically significant PCa was set to 25%, a positive SelectMDx value yielded a 36% probability of correct diagnosis, and a negative value yielded an 11% probability of the wrong diagnosis. When the pre-test probability for diagnosing clinically significant PCa was set to 45%, a positive SelectMDx value showed a 58% probability of correct diagnosis, and a negative value showed a 23% probability of the wrong diagnosis.

#### Sensitivity analysis and heterogeneity sources

The random-effects model was used for sensitivity analysis after the included studies were eliminated one by one. The results are shown in Table 3. The results suggested that no significant changes in the overall value of the combined effects for SelectMDx and the stability were good.

**Table 3:** Sensitivity analysis of included 14 studies.

Study omitted	Estimate (DOR)	[95% Confidence Interval]	
Busetto [29]	4.2989464	2.6399035	7.0006118
Cussenot [41]	5.2260852	3.3197651	8.2270784
Haese [30]	3.9453061	2.4084313	6.4628954
Hendriks [31]	4.0178671	2.3208685	6.9556952
Lendinez-Cano [32]	4.6864419	2.7816806	7.8954921
Maggi [33]	4.1682625	2.4224498	7.1722493
Morote [34]	4.7384415	2.8864396	7.778728
Pepe [35]	4.7909832	2.9169462	7.8690238
Rahnama’i [36]	4.5917959	2.8010027	7.5275149
Roumiguié [37]	4.6701946	2.7909315	7.8148527
Sessine [38]	4.5098972	2.7398882	7.4233584
Shore [39]	4.8971691	3.0219297	7.9360771
Stanton [42]	4.2679458	2.5919459	7.027678
Wysock [40]	4.5794363	2.7311351	7.6785798
Combined	4.5182044	2.7822230	7.3373596

**Fig 5:**
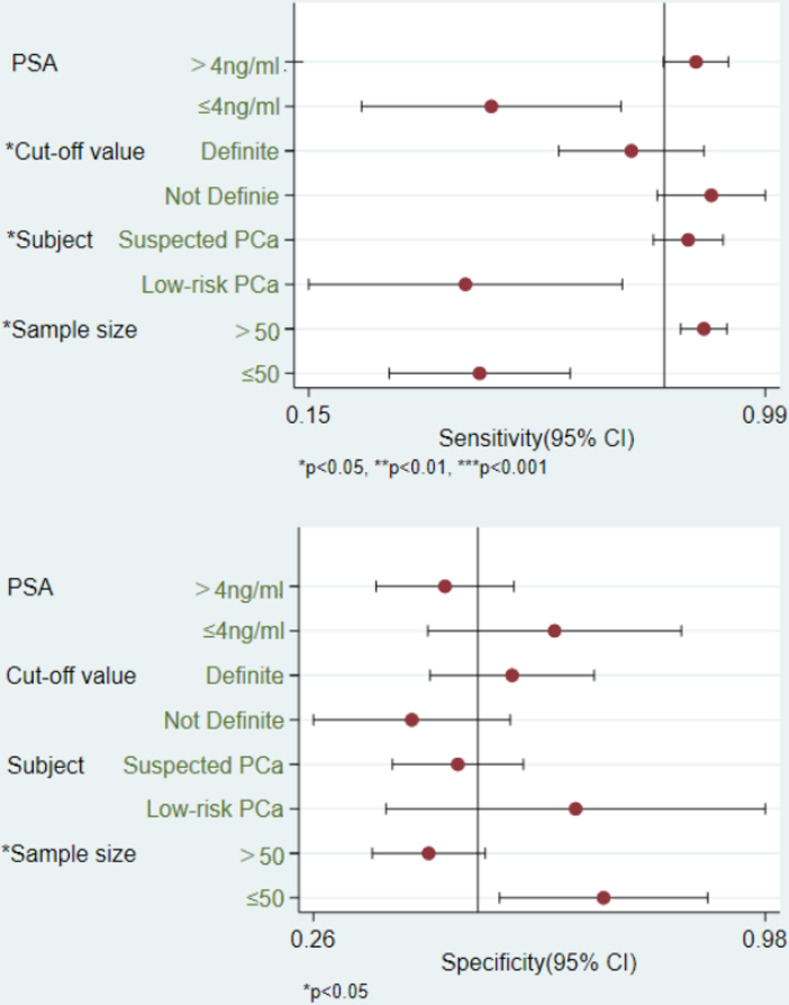
Univariable meta-regression & subgroup analysis.

Median PSA level (>4 ng/ml or ≤4 ng/ml), definite cut-off value, subject (low-risk PCa or patients suspected with PCa), and sample size (≤50 or >50) were included as covariates in a meta-regression analysis (Fig 5). Results showed that median PSA level was not associated with diagnostic accuracy. A definite cut-off was associated with the heterogeneity of sensitivity (P = 0.02). In patients suspected of PCa, the sensitivity of SelectMDx was significantly higher (P = 0.04). The sample sizes of included studies (≤50 or >50) were responsible for the heterogeneity of both sensitivity and specificity (P = 0.02 and P = 0.02, respectively).

**Fig 6:**
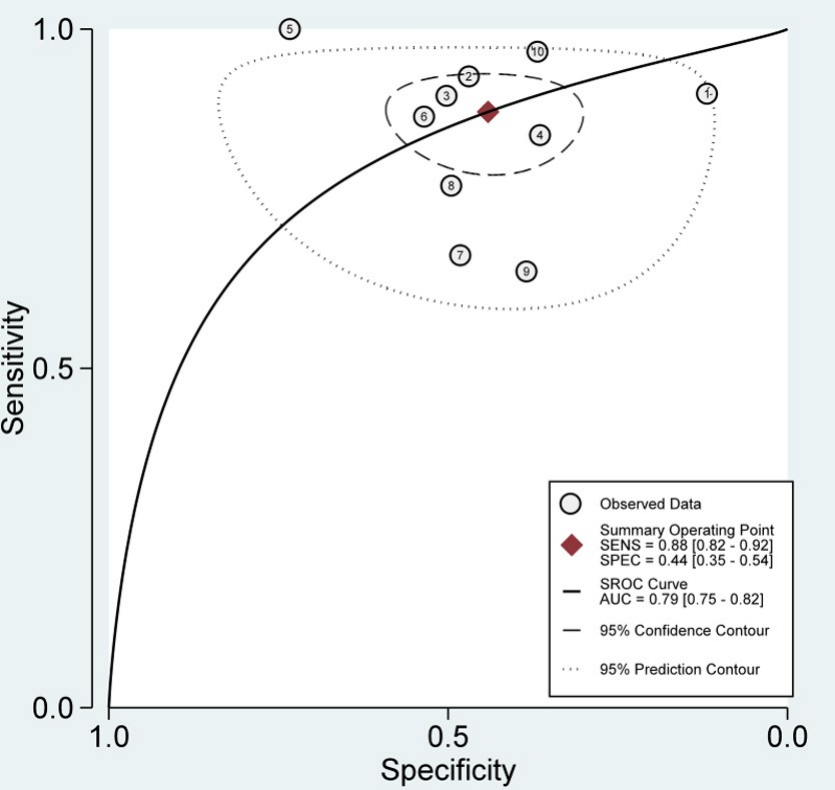
Separate pooled analysis for sample size>50.

A separate pooled analysis for the studies with sample size >50 resulted in a sensitivity of 88% [95% CI, 82%-92%; I2 = 66.87%], a specificity of 44% [95% CI, 35%-54%; I2 = 85.95%] (Fig 6) and an AUC of 79% [95% CI, 75%-82%].

### Publication bias

**Fig 7:**
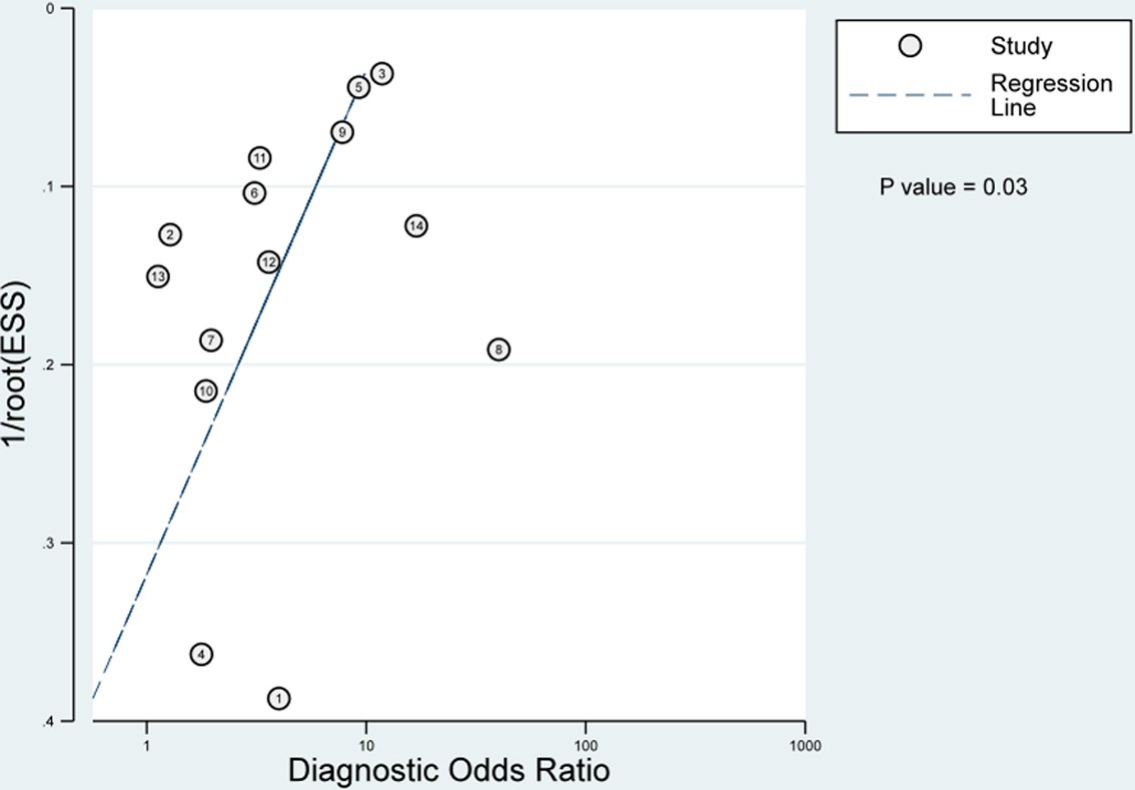
Deek’s funnel plot asymmetry test.

A Deeks funnel plot was constructed to test for publication bias, with statistical significance (P = 0.03) being assessed using Deeks’s asymmetry test (Fig 7).

## Discussion

Given the high incidence of prostate cancer, there is still a large burden of disease both in diagnosis and management [46]. Since the introduction of PSA monitoring in the early 1990s as a screening tool, more and more patients are diagnosed at an early stage [47]. However, not all patients could benefit from PSA screening. Previous research suggested that approximately three-quarters of a million men, who were unlikely to reap a meaningful benefit, received PSA screening [48]. Research shows that men with high-grade PCa have a relatively high probability of dying from PCa within 10 years, while men with low-grade disease have minimal risk [49]. Currently, the challenge is the timely diagnosis and accurate identification of clinically significant PCa.

SelectMDx is a non-invasive diagnostic tool specifically developed for the identification of high-grade PCa (Gleason score≥7). In this systematic review and meta-analysis, we systematically summarized the existing evidence on the diagnostic value of SelectMDx for clinically significant prostate cancer. This meta-analysis showed that SelectMDx has higher sensitivity but lower specificity, with an AUC of 0.73. The overall results indicated that SelectMDx had a certain value in the diagnosis of clinically significant prostate cancer. The twelve included studies were highly heterogeneous and differences in patients and sample size had a statistically significant effect on the diagnostic test accuracy. Stratified results suggested specificity was higher in individuals with low-risk PCa, but the conclusion is limited by the small sample size and the number of studies. As an emerging diagnostic test developed in 2017, some of the research on SelectMDx is still at an exploratory stage, which was characterized by a small sample size and insufficient information on methodology to evaluate the risk of bias [35, 36, 38, 40]. There was considerable heterogeneity of results between studies. Sample size, cut-off value, and selection of participants all affected the heterogeneity. To minimize heterogeneity, future research could report more detailed methodological information on SelectMDx, try to choose globally recognized institution to performed testing or expand the sample size.

A separate pooled analysis also indicated that, in studies with larger sample sizes, SelectMDx has a higher diagnostic value. Future large-scale studies are needed to confirm our results. In future clinical practice, SelectMDx can be used as a tool for the detection of high-grade PCa to avoid unnecessary prostate biopsy and overdiagnosis of indolent prostate cancer. On the other hand, as a diagnostic tool with a high diagnostic value specifically for high-grade PCa, the application of SelectMDx may also help to improve compliance to prostate biopsy for biopsy-negative men in later follow-ups.

There are also many other tests based on serum and urine biomarkers to improve the detection of clinically significant PCa, including the 4-Kallikrein score (4Kscore), Prostate Health Index (PHI), ExoDx Prostate Intelliscore (EPI), and MyProstateScore (MPS) [17, 50–53]. In addition to these biomarkers tests, multiparametric magnetic resonance imaging (mpMRI) of the prostate may also be identified as a useful adjunct for the diagnosis of clinically significant PCa [54]. These diagnostic tools have been validated in various populations and showed some diagnostic value beyond baseline clinical data [55, 56]. However, these tools are not recommended as first-line screening tests by the National Comprehensive Cancer Network (NCCN), which may be because of the small sample sizes and variation of results in current studies [57].

In the course of the research, we observed that a small number of studies have explored the diagnostic accuracy differences between different biomarkers. A study conducted by Wysock *et al*. proposed that when combined with magnetic resonance imaging 4Kscore was superior to the SelectMDx for detecting csPCa. Fiorella *et al*. investigated the performance of SelectMDx and PCA3 in the context of low or very low-risk PCa [40]. The results showed that SelectMDx predicted 5 years of pathological progression free survival with a moderate discrimination ability outperforming PCA3 and the combination of both tests did not improve outcomes [58].

In contemporary years, mpMRI is becoming an integral component in the clinical diagnosis of PCa and has been introduced to the standard of care for the prostate cancer diagnostic pathway [56, 59]. It’s notable that, in our review, we identified several studies in which the combined diagnostic performance of SelectMDx and mpMRI was examined [31, 36, 60]. When the conditional strategy was used, which combined positive results of the SelectMDx test and mpMRI, the biopsy avoidance rate could reach 60%, compared with the mpMRI alone (49%) [31]. In addition, the overdiagnosis of low-grade could also be reduced by 58% with the conditional strategy [31]. However, due to a lack of sufficient data, we were unable to conduct further pooled analysis. In the detection of PCa patients with Gleason score ≥3 + 3, a combination of high-risk SelectMDx and PI-RADS scores of 4/5 could help avoid 87% of biopsies [60]. Future studies may therefore be able to adapt and further develop the combination of novel diagnostic tools to further improve the diagnostic accuracy of high-grade PCa.

### Limitations

This study also has some limitations. Significant heterogeneity was observed among included studies, which made it difficult to interpret the results of this meta-analysis. Secondly, the potential differences in the technical parameter and levels of different institutions may also have an impact on the reliability of results. Furthermore, the funnel plots showed a significant asymmetrical funnel distribution, which indicated the presence of publication bias. Finally, there may be some potential selection bias as only patients who underwent prostate biopsy were included in this study.

## Conclusion

In conclusion, this meta-analysis has shown that the SelectMDx is sensitive and has a certain diagnostic value for the detection of high-grade prostate cancer. This diagnostic tool promises to be further applied in the clinical setting to improve the efficacy of identifying high-grade PCa and bring large health benefits.

## Supporting information

S1 File Search strategies. (DOCX)

S2 File PRISMA checklist. (DOCX)
