# Supplementary material for: A meta-analysis for the diagnostic accuracy of SelectMDx in prostate cancer
Source: PLoS One. 2024 Feb 8;19(2):e0285745. doi: 10.1371/journal.pone.0285745 (PMC10852267; doi:10.1371/journal.pone.0285745)
Supplement: S1 File — (DOCX) [file pone.0285745.s001.docx]

**Search strategy**

**Pubmed:**

#1 "SelectMDx"[Title/Abstract]

#2 "2-Gene mRNA urine test"[Title/Abstract] OR "urinary molecular biomarker-based risk score"[Title/Abstract]

#3 "HOXC6"[Title/Abstract] OR "DLX1"[Title/Abstract]

#4 #1 OR #2 OR #3

#5 "prostate cancer"[Title/Abstract]

#6 "prostate neoplasms"[Title/Abstract] OR "prostatic cancer"[Title/Abstract] OR "PCa"[Title/Abstract]

#7 #5 OR #6

#8 #4 AND #7

**Web of Science:**

#1 TS=(“SelectMDx”OR “2-Gene mRNA urine test” OR “urinary molecular biomarker-based risk score”)

#2 TS=(“HOXC6” OR “DLX1”)

#3 #1 OR #2

#4 TS=(“prostate cancer” OR “prostate neoplasms” OR “prostatic cancer” OR "PCa")

#5 #3 AND #4

, “prostate cancer”, “prostate neoplasms”, and “prostatic cancer” “PCa”

**Embase:**

('SelectMDx'/exp OR 'SelectMDx':ab,ti,kw OR '2-Gene mRNA urine test':ab,ti,kw OR 'urinary molecular biomarker-based risk score':ab,ti,kw OR 'HOXC6':ab,ti,kw OR 'DLX1':ab,ti,kw) AND ('prostate cancer'/exp OR 'prostate cancer':ab,ti,kw OR 'prostate neoplasms':ab,ti,kw OR 'prostatic cancer':ab,ti,kw OR 'PCa':ab,ti,kw)
